# Eelgrass Detritus as a Food Source for the Sea Cucumber *Apostichopus japonicus* Selenka (Echinidermata: Holothuroidea) in Coastal Waters of North China: An Experimental Study in Flow-Through Systems

**DOI:** 10.1371/journal.pone.0058293

**Published:** 2013-03-07

**Authors:** Xujia Liu, Yi Zhou, Hongsheng Yang, Shaoguo Ru

**Affiliations:** 1 Key Laboratory of Marine Ecology and Environmental Sciences, Institute of Oceanology, Chinese Academy of Sciences, Qingdao, People’s Republic of China; 2 Guangxi Institute of Oceanology, Beihai, People’s Republic of China; 3 School of Marine Life Sciences, Ocean University of China, Qingdao, People’s Republic of China; University of Toronto, Canada

## Abstract

Eelgrass ecosystems have a wide variety of ecological functions in which living tissues and detritus may be a food source for many marine animals. In this study, we conducted a laboratory simulating experiment to understand the trophic relationship between the eelgrass *Zostera marina* L and the sea cucumber *Apostichopus japonicus*. A mixture of decaying eelgrass debris and seafloor surface muddy sediments was used as food to feed *A. japonicus*, and then specific growth rates (SGR) and fecal production rates (FPR) were measured. According to the proportion of eelgrass debris, we designed five treatment diets, i.e., ES0, ES10, ES20, ES40, and ES100, with eelgrass debris accounting for 0%, 10%, 20%, 40%, and 100% in dry weight, respectively. Results showed that diet composition had a great influence on the growth *of A. japonicus*. Sea cucumbers could use decaying eelgrass debris as their food source; and when the organic content of a mixture of eelgrass debris and sediment was 19.6% (ES40), a relatively high SGR (1.54%·d^−1^) and FPR (1.31 g·ind.^−1^ d^−1^) of *A. japonicus* were obtained. It is suggested that eelgrass beds can not only provide habitat for the sea cucumber *A. japonicus* but can also provide an indirect food source for the deposit feeder. This means that the restoration and reconstruction of eelgrass beds, especially in coastal waters of China, would be a potential and effective measure for sea-cucumber fisheries, in respect to both resource restoration and aquaculture of this valuable species.

## Introduction

Seagrass beds are estimated to be among the most valuable ecosystems in the world in terms of the value-added services they provide [1]. They are highly productive coastal ecosystems [2], and have important influences on food web support and biogeochemical cycling [3], [4]. They provide food and shelter for a diverse marine fauna, including crustaceans, fish and mollusks, and serve as a nursery habitat, providing predation refuge for juvenile animals [5]–[7].

Seagrasses act as a direct food source for herbivores and also enter detrital food webs [8]. In seagrass ecosystems, some marine herbivores can ingest seagrass leaves, epiorganisms and large algae directly [9], [10], while filter feeders utilize phytoplankton, but there are a few detritus feeders that feed on detritus after the eelgrass decomposes [11], [12]. The nutrients produced by decomposition can then be utilized by the primary producers, which then become an important part of the detrital food chain and facilitate the cycling of nutrients. Some researchers point out that seagrasses contribute to secondary production mainly by the detrital food web [8], [13].

Seagrass beds can change fluid dynamics, accelerate the sedimentation of suspended particulates, stabilize the sediment and improve water quality, with consequent benefits to the growth of other aquatic organisms [14]. However, seagrass beds have declined dramatically worldwide, partly due to natural disasters but largely due to an increase in human activities [15]–[17].

The eelgrass *Zostera marina* L. is a kind of perennial marine angiosperm belonging to the class Monocotyledoneae and order Helobiae. It is distributed worldwide in the intertidal and subtidal zones of shallow seas. It grows in muddy or sandy substrate, and in areas with reduced water flow and good water clarity. It occurs in the temperate coastal areas of Shandong, Hebei and Liaoning provinces in north China [18]. In China eelgrass has decreased greatly since the 1970s, owing to human-induced habitat deterioration. Increasing human activities are generally considered to be responsible for this reduction. Due to the low economic value of eelgrass itself, eelgrass meadows have not been treated in a reasonable way, and have even been removed completely, as a fouling organism. However, on a more positive note, the public understanding of the importance of eelgrass in ecosystem functioning has been enhanced in recent years. There has been an increase in research on eelgrass in China [19]–[21], but few studies on eelgrass restoration.

The sea cucumber *Apostichopus* (*Stichopus*) *japonicus* Selenka, belonging to the phylum Echinodermata, class Holothuroidea, and order Aspidochirotida, is an epibenthic, temperate species and is mainly distributed in the shallow seas of the north Pacific Ocean [22]. The deposit feeder *A. japonicus* is an important fishery and aquaculture species with a high commercial value, especially in the Asian market. It inhabits sandy, muddy sand substrate and rocky areas, especially where the bottom has flourishing aquatic plants, such as seagrass and seaweed. Deposit-feeding Stichopodid sea cucumbers ingest sediment bearing organic matter, including bacteria, protozoa, diatoms, and the debris from plants or animals [23]–[25]. Recent studies have shown that food residue and feces of marine animals are also a food source for sea cucumbers [26]–[33]. With a high global demand for sea cucumbers, especially for *A. japonicus* as Bêche-de-mer in the Asian market, the increasing harvest pressure on natural populations has created severe overfishing throughout the world [34]–[36].

Swan Lake is a small cove located in Weihai, North China, where the area of eelgrass is now expanding due to effective conservation and restoration measures. Sea cucumbers inhabit the eelgrass meadow of the cove in high densities. They were even more abundant 30 years ago, when eelgrass covered almost the whole cove; and there is a vivid description on the abundance of sea cucumbers in local historic accounts, i.e. one step several individuals, that means an extremely high density of ca.10–30 ind·m^−2^.

Although *A. japonicus* always occurs abundantly in eelgrass-rich meadows in temperate northern coastal areas of China; yet it has never been reported whether eelgrass detritus can act as a food source for sea cucumbers or not. In this study, decaying eelgrass debris and muddy sediment were mixed as food to feed *A. japonicas*. The ingestion and growth of the animals were then determined, in order to understand the importance of eelgrass meadows for *A. japonicus*, and thus clarify the importance of eelgrass-meadow restoration. It is suggested that eelgrass-meadow restoration is imperative and could not only be of huge ecological benefit, but also of potential economic value.

## Materials and Methods

### Ethics Statement

The collecting of the decaying eelgrass leaves and the invertebrate *Apostichopus japonicus* from Swan Lake of Weihai was permitted by Peiliang Wang, manager of Mashan Group Co. Ltd. No specific permit was required for the collecting of the sediment from Jiaozhou Bay, Qingdao, where is not privately owned or protected. Ethical approval was not required for this study because no endangered animals were involved. However, specimen collection and maintenance were performed in strict accordance with the recommendations of Animal Care Quality Assurance in China.

### Experimental Materials

Eelgrass debris used in this study was from decaying eelgrass leaves. The decaying dark colored eelgrass leaves were collected from Swan Lake of Weihai. Muddy sediment was obtained from the non-vegetated seafloor surface in the inshore area of Jiaozhou Bay, Qingdao. Both decaying eelgrass leaves and sediment were dried at 65°C, ground and sieved using 0.18 mm mesh. The specimens of *A. japonicus* used in this study were also collected from the cove of Swan Lake. Sea cucumbers were transported to the laboratory where they were acclimatized for a week, after which an experiment was carried out using 20 PVC boxes, with each box measuring 30×40×30 cm in size.

### Experiment Design

Mixtures of *Z. marina* debris and sediment were used to feed *A. japonicus*. According to the proportion of *Z. marina* debris, 5 diet treatments in quadruplicate were designed, i.e., ES0, ES10, ES20, ES40, and ES100, with eelgrass debris accounting for 0%, 10%, 20%, 40%, and 100% in dry weight, respectively. The chemical composition of each diet was analyzed in 3 replicates. Each diet treatment involved 4 aforementioned PVC boxes, and each box contained 2 individuals of *A. japonicus* with initial wet body weights of 25.12±5.79 g·ind.^−1^. The experiment was conducted from April 22nd to June 8th, 2009 in the laboratory at the Institute of Oceanology, Chinese Academy of Sciences, Qingdao, P. R. China. During the experiment, specific growth rates and fecal production rates of A. japonicus were measured.

### Rearing Conditions

During the experiment, one-half volume of water was exchanged every day to ensure good water quality. Seawater used in the experiment was filtered by a composite sand filter. Seawater conditions were pH 7.8–8.2; salinity 30–32 ppt; dissolved oxygen was maintained above 5.0 mg L^−1^.

### Feeding Methods

According to the food consumption observed during the first week, between 15 and 35 g of food per week was provided to ensure an excess of food. Eelgrass debris and sediment were well mixed, slightly wetted, stirred to a lumpy consistency, and then evenly scattered onto the bottom of each PVC box. Uneaten food was removed every 3 days.

### Sample Collection and Measurement

The initial and final wet body weight of *A. japonicus* was measured. Animal feces were collected by siphon every 1–3 days, depending on the amount of produced feces. For estimation of assimilation efficiency (AE) of *A. japonicus*, fresh feces produced within 6 h were collected. Fecal samples were dried at 60°C to constant weight and preserved for further analysis. Subsamples of the dried food and feces were used to estimate organic material (OM) by combusting (500°C for 3 h) dried and pre-weighed samples. Subsamples were treated with HCl vapor for 6 h to remove carbonates for analyzing organic carbon (OC) and nitrogen (ON) with a Perkin Elmer Model 240c CHN analyzer standardized with acetanilide.

### 2.6. Data Calculation and Statistical Analysis

Specific growth rate (SGR; %·d^−1^), fecal production rate (FPR; g·ind.^−1^ d^−1^)), and assimilation efficiency (AE; %) were calculated as follows:
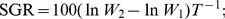


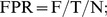


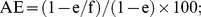
where, *W_1_* and *W_2_* are initial and final wet body weight of *A. japonicus* in each PVC box; *T* is the duration of the experiment; *F* is the dry weight of feces; *N* is the number of *A. japonicus* in each PVC box; *e* and *f* are organic matter percentages in fresh feces and food respectively.

Values are presented as mean±SD, n = No. of replicates. Differences between treatments were tested using one way analysis of variance (ANOVA). Prior to analysis, data were examined for homogeneity of variances (F test). Differences were considered significant at a probability level of 0.05. Statistics were performed using software SPSS 16.0.

## Results

In this experiment, the organic contents of food in the five diet treatments were 5.6%, 7.6%, 10.9%, 19.6%, 39.9%, respectively; and organic matter in the feces of *A. japonicus* was lower than that in the food; and organic carbon and nitrogen were the same (Table 1).

**Table 1:** Content of organic matter in food and feces for five diet treatments.

Diettreatments	Content of organicmatter in food (%)	Content of organiccarbon in food (%)	Content of organic nitrogen in food (%)	Content of organic matter in feces (%)	Content of organic carbon in feces (%)	Content of organic nitrogen in feces (%)
ES0	5.6	0.91	0.04	−	−	−
ES10	7.6	2.65	0.18	6.59±0.48	2.48±0.35	0.16±0.03
ES20	10.9	4.32	0.30	9.51±0.76	3.96±1.08	0.25±0.07
ES40	19.6	7.75	0.58	15.61±0.98	7.29±0.65	0.49±0.09
ES100	39.9	17.86	1.36	34.09±2.10	16.62±2.07	1.09±0.20

Notes: “−”, undetected; SD, standard deviation.

At the beginning of the experiment, there were no significant differences in wet body weights of *A. japonicus* between the five treatments (F = 1.20, df = 39, p* = *0.33) (Table 2). By contrast, at the end of the experiment, final wet body weight of *A. japonicus* in treatment ES0 was significantly lower than that in other treatments containing eelgrass debris (all p<0.05). And final wet body weight in treatment ES40 was significantly higher that that in treatments ES10 and ES20 (both p<0.05).

**Table 2:** Initial and final wet weight (g·ind.^−1^) of *A. japonicus* for five diet treatments.

Wet weight (g·ind.^−1^)	Diet treatments				
	ES0	ES10	ES20	ES40	ES100
Initial	26.79±3.05	22.78±.4.34	25.86±7.94	24.04±3.93	27.12±7.59
Final	20.68±2.76^a^	26.17±3.78^b^	30.10±7.36^bc^	36.35±4.09^d^	36.97±8.30^cd^

Note: values with different letters in the same row were significantly different from each other (n = 4, *p*<0.05).

**Figure 1:**
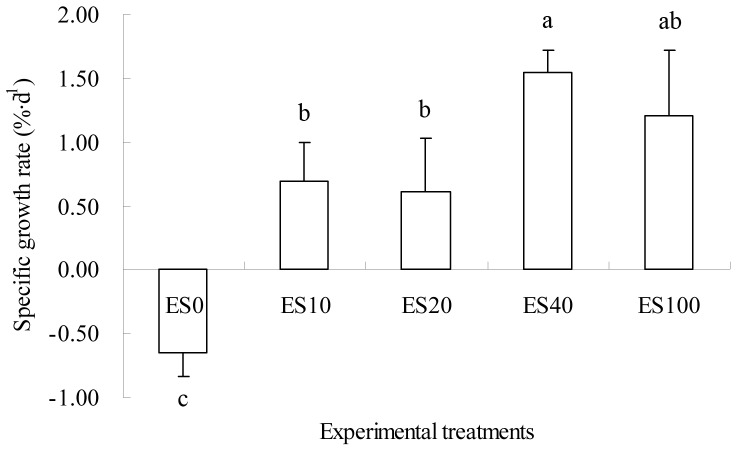
Specific growth rates (SGR; %·d^−1^) of *Apostichopus japonicus*. Means (n = 4) with different letters denoting significant differences (*p*<0.05), and bars representing standard deviations of the means.

SGR of *A. japonicus* in treatment ES0 with a negative value was significantly lower than that in other treatments (all p<0.05) (Fig. 1). And SGR of *A. japonicus* in treatment ES40 was significantly higher than those in treatments ES10 and ES20 (F = 24.08, df = 7, p* = *0.003; F = 16.57, df = 7, p* = *0.007, respectively). No significant differences were found between the other treatments ES10, ES20 and ES100 (all p>0.05).

**Figure 2:**
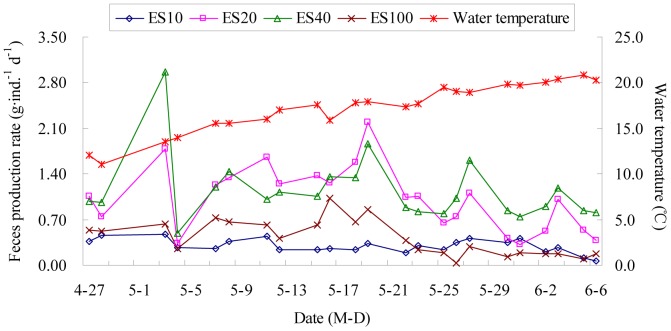
Variation in Fecal production rates (FPR; g·ind. ^−**1**^ **d** ^−**1**^ **) of** ***Apostichopus japonicus*** **during the experimental period.**

**Figure 3:**
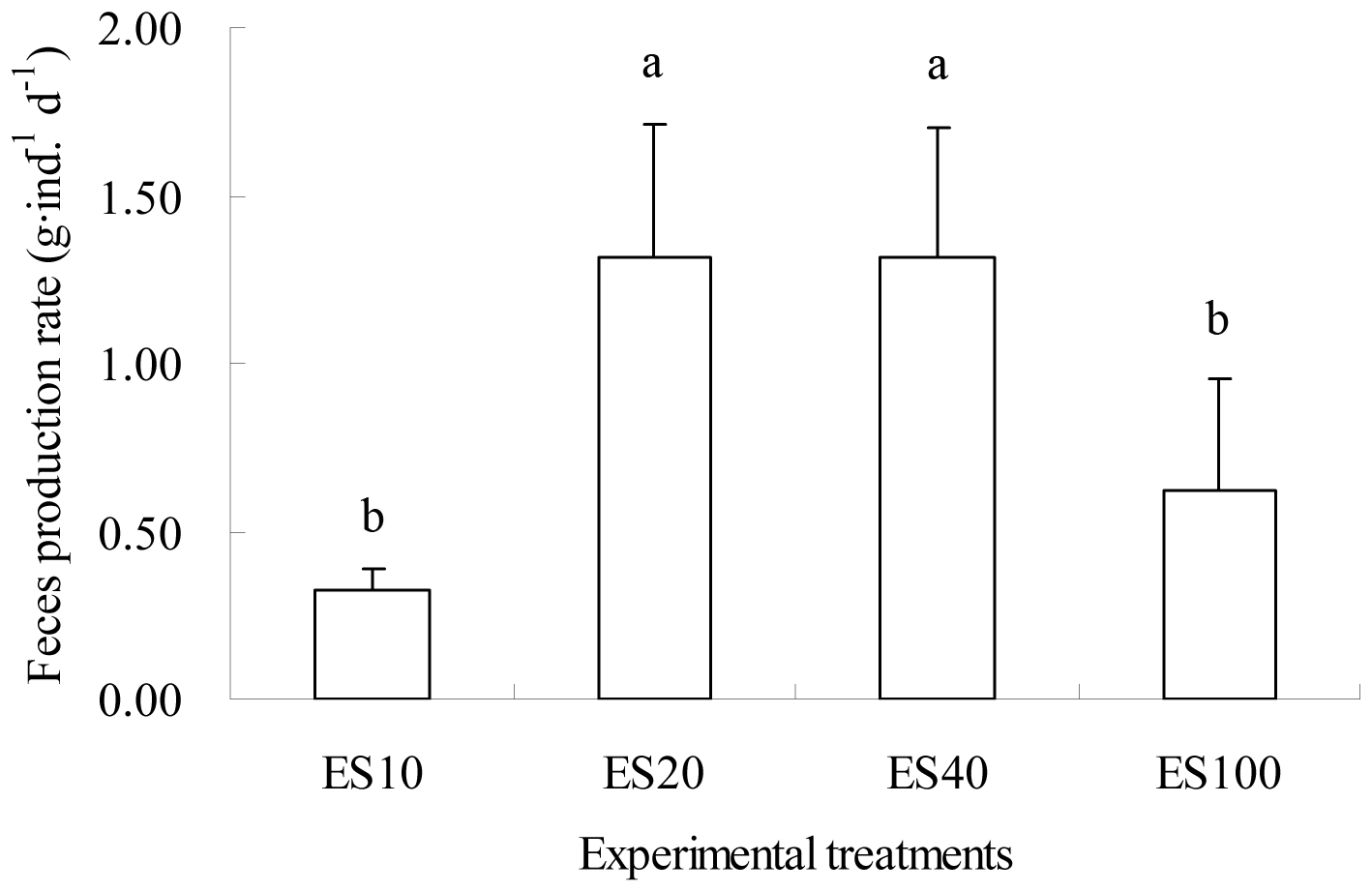
Mean fecal production rates (FPR; g·ind. ^−**1**^ **d** ^−**1**^ **) of** ***Apostichopus japonicus*** **during the experimental period.** Means (n = 4) with different letters denoting significant differences (*p*<0.05), and bars representing standard deviations of the means.

Fig. 2 shows variation in fecal production rates (FPR; g·ind.^−1^ d^−1^) of *A. japonicus* during the experimental period. Sea cucumbers in treatment ES0 had hardly ingested food during the experiment period, thus only fecal production rates in other treatments were considered comparable with each other. Generally, FPRs in treatments ES20 and ES40 were significantly higher than those in treatments ES10 and ES100 (all p<0.05; Fig. 3).

**Figure 4:**
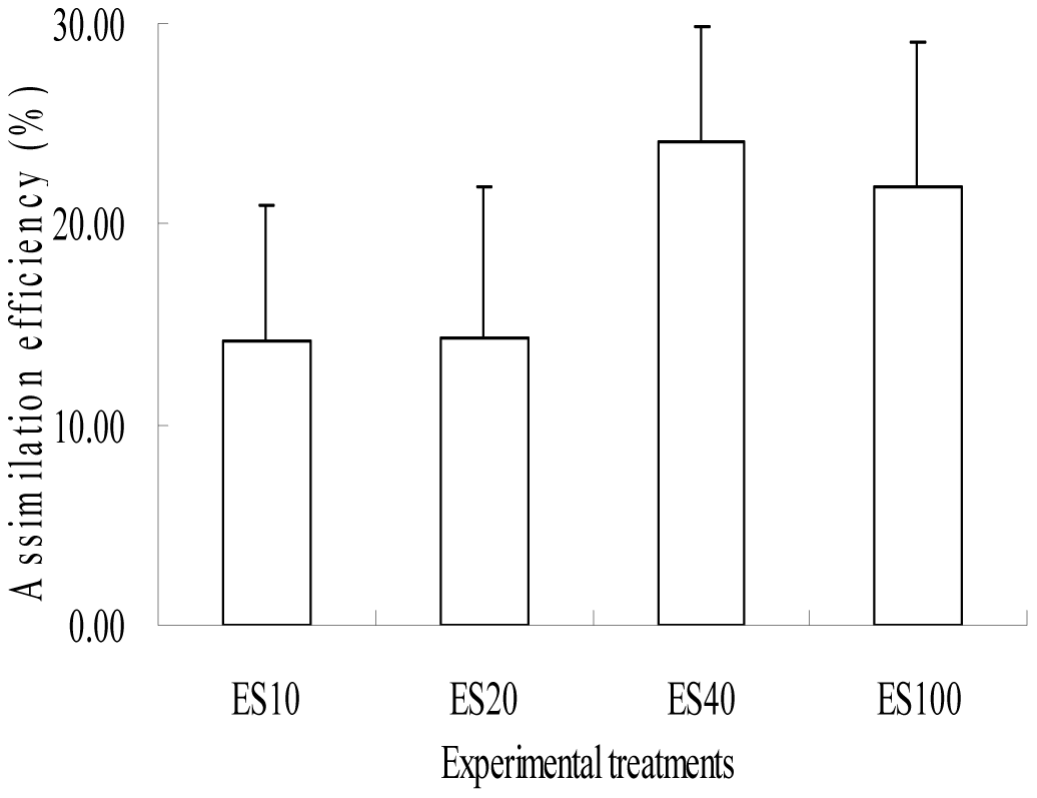
Assimilation efficiency (AE) of *Apostichopus japonicus*. Bars represent standard deviations of the means.

Assimilation efficiency (AE) of organic matter by *A. japonicus* had no significant difference in four experimental treatments (Fig. 4), with mean AE being 14.2%, 14.3%, 24.2%, and 21.8%, respectively (all p>0.05).

## Discussion

In northern inshore areas of China, many other animals inhabit the eelgrass ecosystems in which *A. japonicus* is abundant. This study showed that decaying eelgrass debris could act as an important food resource for *A. japonicus*.

In this experiment, SGR of *A. japonicus* in the five treatments were significantly different. SGR was only −0.65% d^−1^ in the pure sediment diet (treatment ES0), which was significantly lower than that in the mixed diets of *Z. marina* debris and sediment. In contrast, the mean SGR in treatments ES10, ES20, ES40 and ES100 were 0.70%·d^−1^, 0.61%·d^−1^, 1.54%·d^−1^, and 1.20%·d^−1^, respectively (Fig. 1). The occurrence of negative growth of *A. japonicus* fed with pure sediment might be due to the lower organic matter content (5.6%) of the diet, which might not satisfy the need for growth. The highest SGR of *A. japonicus* occurred in treatment ES40 with organic matter content being 19.6% and higher than that in ES0, ES10, and ES20; while in the pure eelgrass-debris diet (ES100) with organic matter content as high as 39.9%, the SGR was not significantly higher than that in treatment ES40 (p>0.05). Sea cucumbers are deposit feeders, and clay sediment is an important component of ingested material, which is supposed to be helpful for the digestion of food [37].

During the experiment, food was scattered on the bottom of the PVC boxes, thus residual food was difficult to collect. Nevertheless, to some extent, the fecal production rate can be substituted for the ingestion rate. In the natural ecosystem, in cases where sediment of low nutritional value was ingested by deposit feeders, internal appetite regulation would actively work to increase food ingestion [38]; and when food quality becomes better, in certain seasons, deposit feeders would decrease food ingestion. The same phenomenon was also found in other echinoderms. Otero-Villanueva et al. found in the regular echinoid *Psammechinus miliaris* that the lowest ingestion rate was related to high energetic food [39].

Generally, *A. japonicus* feeds on sediments containing organic matter, which includes microorganisms and the detritus of plants or animals. Results in this experiment showed that *A. japonicus* could use eelgrass detritus as a food resource. According to the present study, a mixture of *Z. marina* debris and seafloor muddy sediments with an organic content of 19.6% (ES40), could lead to a better growth effect.

Some researchers pointed out that the appropriate temperature for the growth of *A. japonicus* is 5–20°C, while the optimum is 10–16°C [40]–[42]. During the experiment, the water temperature ranged from 13.5–20.8°C (Fig. 2). With an increase in water temperature to 18°C, *A. japonicus* gradually went into an aestivation state, and the FPR of *A. japonicus* reduced rapidly (Fig. 2). Before entering into aestivation, the FPR of *A. japonicus* was higher, which were 0.33 g·ind.^−1^ d^−1^, 1.31 g·ind.^−1^ d^−1^, 1.31 g·ind. ^−1^ d^−1^, 0.63 g·ind.^−1^ d^−1^ for treatments ES10, ES20, ES40, and ES100, respectively (Fig. 3). According to the experiment the water temperature had an effect on the SGR and FPR of *A. japonicus* which fed on the mixed food containing eelgrass detritus and seafloor surface sediment. When the water temperature was between 13 and 17°C, *A. japonicus* grew faster.

In natural eelgrass ecosystems, deposit feeders feed on organic detritus after eelgrass decomposition, which not only accelerates the cycling of matter but also promotes the health and stability of the ecosystem structure. Until now eelgrass meadows in Shandong coastal waters with water depth 2–6 m have deteriorated badly, and some eelgrass meadows have even disappeared [43]. For the restoration of the declining natural resource of *A. japonicus,* it is suggested that the degraded eelgrass meadows should be restored in the northern inshore areas of China.

In conclusion, *A. japonicus* can feed on eelgrass detritus, and a mixture of *Z. marina* decaying debris and seafloor surface sediments, with an organic content approximately 20%, can meet their nutritional needs. Eelgrass meadows can not only provide habitat for *A. japonicus*, but also provide a food source for the animal, which is significant for eelgrass ecology, and the resource restoration of *A. japonicus*, especially in coastal waters of China. Therefore eelgrass restoration in China would bring inestimable ecological benefits and increased economic value. Also, this study implied that dense *A. japonicus* might play an important role in processing organic detritus-rich sediments and accelerates the cycling of matter in eelgrass-vegetated coastal ecosystems, on which further study is necessary to be conducted in the future.
